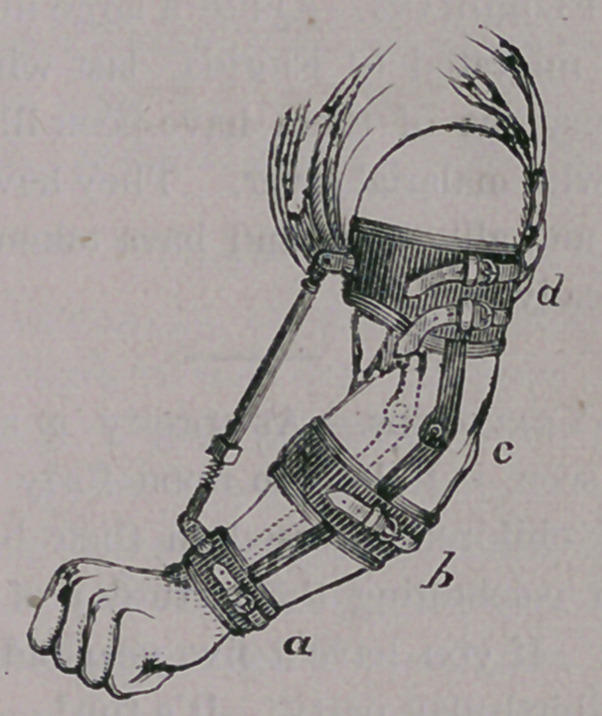# Anchylosis

**Published:** 1876-07

**Authors:** 


### ANCHYLOSIS.

This term is used by surgeons to indicate a stiff or immovable joint. It is likely to happen to any of our joints, but most frequently occurs to the elbow or knee, usually the most inconvenient locality. A stiff joint is likely to occur from any cause that produces inflammation in the synovial membrane, (the membrane which surrounds the joint and secretes the fluid which lubricates it), as from blows, wounds, sprains, dislocations or fractures near the joint. The majority of cases are found to exist from fractures in or about the joints, and are the most stubborn to treat, for the reason that when the joint is necessarily kept quiet for several weeks, the synovial membrane becomes dry and stiff, and, irritated by the fracture near by, puts on an inflammation, when plastic or adhesive matter is thrown out in the joint, which not only fills up the natural cavity in which the head of the bone moves, but causes strong fibrous bands to unite the heads of the bones in such a manner as to frequently lock all movements in the parts, entirely. In time, if the efforts of the surgeon are not successful to break up these adhesions and allay inflammation, the deposit in the joint becomes bone-like, uniting with the ends of the bones forming the joint, when complete anchylosis occurs, for which no remedy exists.

As soon as the fracture is sufficiently healed, the surgeon applies himself to carefully moving the joint, and to allay all inflammation that may exist in the parts. Daily movements of the joint are caused to be made, and later, an apparatus applied, similar to the one here shown.

If the stiffness is in the knee joint, well padded and snugly fitting bands, surround the limb, above and below the knee—are united by strong, steel braces, which are joined at the knee, and rendered movable by a cog-wheel arrangement, turned with a key, as seen in the illustration. This apparatus and its object will be at once understood by studying our engraving.

For the elbow joint, a similar arrangement is used, the power being applied by means of the screw shown in the cut.

The padded bands support the recently fractured and therefore weakened bones, while the screw power breaks up the attachments formed in the joints, and finally, after much time and patient perseverance, restores the movement of the joint again.

In the more severe cases, where fracture exists *in* the joint, or the inflammation has run so high as to cause a permanent lock, then, of course, all movement of the joint is lost and the surgeon must content himself in placing the limb in such a position as will be most convenient for the patient. In the case of the knee, the limb is set slightly bent, and not straight or at an acute angle. In the elbow, it is best to set it at nearly a right angle, as the patient can then reach the mouth with his hand and will find this position most convenient for the usual occupations of life. These positions can be obtained even after the joints are permanently locked, by drilling into the bone, bending or breaking it to the proper angle, and fixing it there until a new union is obtained.

---

Washington's false teeth are to be exhibited at the Centennial, in company and contrast with the finest dental work of New York. The wonder is, they say, that any man ever held them in his mouth five minutes. The teeth are bits of bone, scarcely trying to look like teeth, attached to gold plate, with strips riveted across to strengthen the teeth in place; while coiled wire at the end of the jaws makes a spring, and assists in opening and closing the machine.

## Figures and Tables

**Figure f1:**
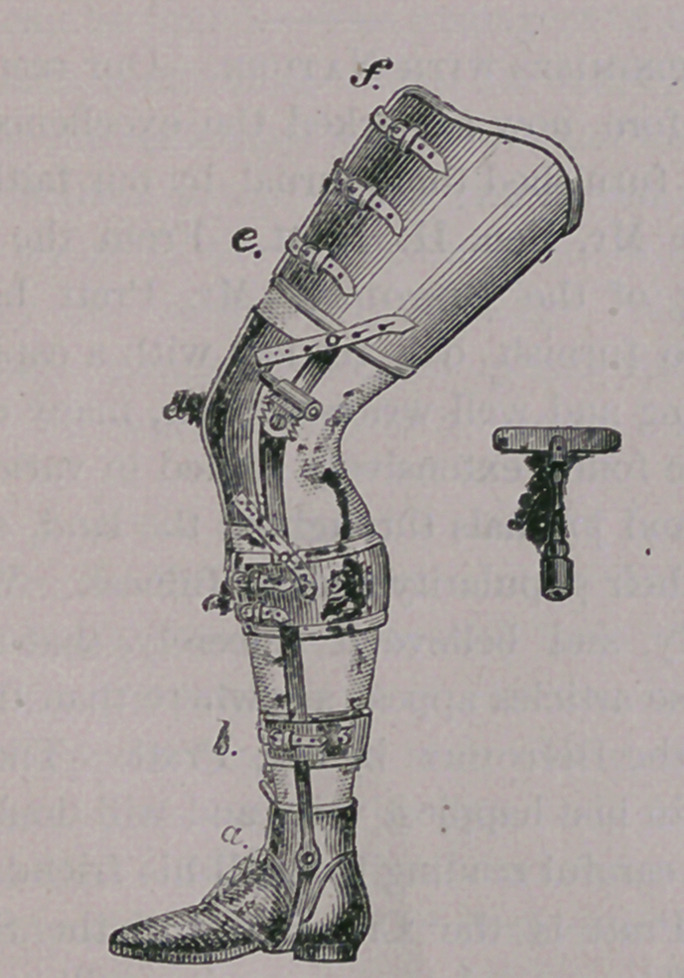


**Figure f2:**